# Pregabalin Alleviates Anxiety and Fear in Cats during Transportation and Veterinary Visits—A Clinical Field Study

**DOI:** 10.3390/ani13030371

**Published:** 2023-01-21

**Authors:** Terttu Lamminen, Mira Korpivaara, John Aspegrén, Clara Palestrini, Karen L. Overall

**Affiliations:** 1 Research &Development, Orion Corporation Orion Pharma, 02100 Espoo, Finland; 2 Department of Veterinary Medicine, University of Milan, 20060 Lodi, Italy; 3 Department of Health Management, Atlantic Veterinary College, University of Prince Edward Island, Charlottentown, PE C1A 4P3, Canada

**Keywords:** feline, pregabalin, anxiety, fear, veterinary visit, clinical examination, transportation

## Abstract

**Simple Summary:**

Cats are often anxious during travel and veterinary visits which can lead to a lack of veterinary care. In this study, a novel pregabalin 50 mg/mL oral solution was tested in 209 cats suffering from anxiety. The cats were given either flavored pregabalin solution or an identical placebo solution without pregabalin 90 min before transporting them in a car for at least 20 min to a veterinary clinic. The effect of the treatment during transportation was evaluated by the cat owner and during clinical examination by the veterinarian. Neither the cat owner nor the veterinarian knew which treatment the cat had received. Both travel- and veterinary-visit-related anxiety were significantly decreased in cats that had received pregabalin. Treatment was well tolerated. Only few cats showed slight incoordination and tiredness for a short time. The owners found a small volume o flavored oral solution user-friendly. It was also well accepted by the cats. This study showed that a single oral dosage of the novel pregabalin solution alleviates anxiety and fear related to transportation and veterinary visits in cats, thus aiding both owners and veterinarians by enabling cat-friendly handling and improving the welfare of cats in stressful situations.

**Abstract:**

Cats frequently suffer from anxiety related to travel and veterinary visits. One sequela is avoidance of veterinary visits and lack of adequate veterinary care. The objective of this study was to test clinical efficacy and safety of a novel formulation of a pregabalin 50 mg/mL oral solution for alleviation of anxiety and fear in cats during transport and veterinary visits. A total of 209 client-owned cats were given either a flavored pregabalin oral solution at the dosage of 5 mg/kg (n = 108) or an identical placebo (n = 101) approximately 90 min before placing them into the carrier and transporting them in a car for at least 20 min to a veterinary clinic. The treatment effect using a 5-point numerical rating scale was evaluated during transportation by the owner and during clinical examination by the veterinarian, both blinded to the treatment. In addition, to verify the owner assessment, an external expert blinded to the treatment and owner assessment evaluated the transportation video recordings using the same rating scale as the owner. Pregabalin 5 mg/kg statistically significantly decreased both travel- (*p* < 0.01) and veterinary-visit- (*p* < 0.01) related anxiety compared to the placebo. The external expert’s evaluation was in agreement with the owners’ assessment confirming the treatment effect during transportation (*p* < 0.01). Treatment was well tolerated with only a few cats showing transient slight incoordination and tiredness. The flavored oral solution formulation with a small dosing volume of 0.1 mL/kg was found by the owners to be user-friendly and was well-accepted by the cats. This study demonstrated that a single oral dosage of the novel pregabalin oral solution alleviates anxiety and fear related to transportation and veterinary visits in cats, thus providing practical aid for both owners and veterinarians to enable cat-friendly handling and improving the welfare of cats in situations they often perceive as very stressful.

## 1. Introduction

Anxiety and fear associated with transportation and veterinary visits is a well-known challenge among cat owners [1,2,3,4]. Based on the results of a cat owner survey by Mariti et al. (2016) [5], most cats show impaired welfare during all stages of a clinic visit: before entering the waiting room, moving to the examination room, on the examination table, and after returning home. Distress worsens with every further experience and has a compounded negative effect on traveling and handling in other situations [5]. As many cats aggressively resist being placed into a carrier and show signs of distress when transported and during veterinary visits, many cat owners defer taking their cat to the veterinarian.

According to a veterinary care usage study by Volk et al. (2011) [2], 40% of cats had not been seen by a veterinarian within the past year, compared to only 15% of dogs. Similar results are shown in another survey, in which 44.9% of cat owners did not take their cats to a veterinarian, despite the recommendation of an annual preventive care visit [6]. Therefore, cats are likely to be more seriously ill before veterinary care is sought.

Pregabalin is a structural analogue of the neurotransmitter gamma-aminobutyric acid (GABA) and binds to the alpha-2-delta subunit of the voltage-dependent calcium channel in the central nervous system [7]. It decreases the release of glutamate and monoamine neurotransmitters involved in the pathophysiology of anxiety [8,9]. At the brain level, attenuation of fear-related activation of the amygdala and anterior insular cortex contributes to the anxiolytic effect of pregabalin [10,11]. In rodent models, pregabalin has shown dose-dependent anxiolytic-like effects [11,12,13]. In a pilot study in client-owned cats, good clinical safety and a significant decrease in signs of anxiety and fear associated with car transportation was reported [14].

Two pharmacokinetic studies of pregabalin in laboratory cats have been published [15,16] showing good absorption and bioavailability, as well as a linear pharmacokinetic profile. Lamminen et al. (2022) [16] reported the bioavailability of 94%, mean maximum plasma concentration of 10.1 µg/mL reached between 0.5 and 1 h, area under the curve of 129 h*µg/mL, and a mean half-life of 14.7 h after administration of the oral solution formulation used in this clinical study with a dose 5 of mg/kg. No safety concerns were reported in healthy laboratory cats.

The objective of this study was to confirm clinical efficacy and safety with the newly developed flavored oral solution formulation in cats showing signs of distress, anxiety, and/or fear during transportation and veterinary visits.

## 2. Materials and Methods

The study was a randomized, double-blind, placebo-controlled, parallel-group, multicenter clinical field study conducted at 22 veterinary clinics in five European countries (Finland, Germany, Hungary, Ireland, and Portugal) between September 2018 and May 2019. The randomization of the study treatment was made before the study started by an independent randomization expert using computer software. The investigators, owners, external expert, and the sponsor representatives were all blinded to the study treatment. The investigators were licensed veterinarians who were willing to participate and able to recruit suitable patients from their patient populations to the study. These veterinarians were trained for the study procedures and their working time used for the study tasks was financially compensated. 

The study was conducted in compliance with Good Clinical Practice (GCP) as defined by the Veterinary International Conference on Harmonization (VICH) Guideline (GL) number 9. The GCP is an acknowledged international ethical and scientific quality standard, and gives assurance about the integrity of the data and animal welfare. The General Data Protection Regulation was fully followed during the study.

The clinical trial application was approved by the competent regulatory authority of each country, and the study protocol was written in accordance with animal welfare standards and requirements. Owner informed consent was obtained in writing from cat owners prior to enrollment. Owners were informed that cats would randomly be assigned to placebo and treatment groups, that all cats would receive physical and laboratory examinations as part of their participation in the study, and possible risks associated with the use of sedatives, if required, were explained. The owners were permitted to withdraw their cat from the study for any reason, at any time. Owners acted as rapporteurs and assessors of the cats’ behaviors for parts of the study as described below, but no data were collected on the owners separate from that for the guardianship of the cats. The health, welfare, treatment, and care of the study animals were ensured by veterinary supervision at each participating clinic and also monitored by sponsor personnel not affiliated with the study site, but trained as clinical study monitors, to ensure humane care of the study animals according to GCP standards.

### 2.1. Animals

Client-owned cats were recruited by the investigators from clientele of their participating veterinary clinics and through advertisements in social media. Cats of any age were eligible to participate to the study if they had a history of being stressed, anxious, and fearful when transported by car and during veterinary visits. They could enter the study after being assessed by a veterinarian as healthy or with mild systemic disease (American Society of Anesthesiologists class I or II). Additionally, cats enrolled were required to score 3–5 at the screening in the owner’s assessment of transportation (Table 1) and 3–5 in the investigator’s assessment of the ability to perform clinical examinations (Table 2). Cats were excluded from participating if they were being treated with other psychoactive medications, homeopathic remedies, pheromonal products, supplements, or a special diet to control anxiety. Other reasons for exclusion were pregnancy, lactation, concurrent participation to any other clinical study, and any other condition or situation which could disturb the conduct of the study, for example, owner’s inability to administer the study treatment, make video recording, or transport the cat in a car.

Since these visits either were annual health visits or mimicked them, all cats received a physical and laboratory examination as part of their participation in the study. All cats were monitored for clinical safety of the study treatment (active or placebo) that they received, as pregabalin was not yet approved for this target species.

### 2.2. Treatments

At screening, cats were given tap water orally with a syringe to mimic study procedures, and baseline assessments were performed. Eligible cats were randomly assigned in a 1:1 ratio to receive either a single 5 mg/kg dose of flavored pregabalin oral solution (Bonqat® 50 mg/ml, Orion Corporation, Espoo, Finland) or a placebo. To ensure blinding, the study treatments were identical in color and odor with the same small dosing volume of 0.1 mL/kg. The study treatments were given at home by the owner who was trained to administer the study product.

### 2.3. Assessments

**Figure 1:**
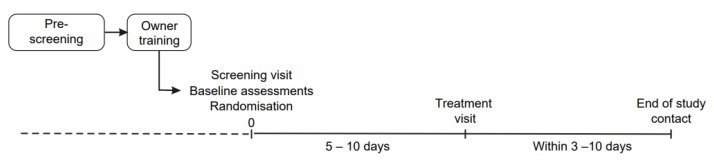
Study design of the clinical field study.

The assessments were done both at screening and treatment visits that were conducted at the interval of 5–10 days (Figure 1). Study treatment (or water at the screening visit) was administered 90 ± 15 min before the cats were placed into a carrier and transported in a car for at least 20 min to the veterinary clinic. A video was recorded during the car transportation. At the clinic, a standardized clinical examination (Table 3) was performed by the investigator, who was the participating clinic veterinarian. The clinical examination was designed to correspond to and coincide with a routine annual health check. To ensure adequate patient population and reliable results, all procedures and assessments were performed in a similar manner at both the screening and treatment visits.

### 2.4. Primary Efficacy Variables

The two primary efficacy variables were the owner’s assessment of the treatment effect during transportation and the investigator’s assessment of the treatment effect during clinical examination. A 5-point scale was used for both variables (Table 1 and Table 2).

### 2.5. Secondary Efficacy Variables 

As secondary efficacy variables, the owners assessed the ability to place the cat into the carrier using a similar 5-point scale. Additionally, the owners assessed signs of stress, anxiety, and/or fear (vocalization, abnormal activity, resistance, destructive behavior, escaping/hiding, withdrawn/crouching, freezing/decreased motor activity, urination, defecation, vomiting, panting, continuous licking, scaling, salivation, sweating paws). Each sign was rated for the extent to which it was expressed according to Table 4. Rating occurred at several time points: during transportation, clinical examination, at home just after opening the carrier, and 1 and 3 h after coming home. The sum of signs of stress, anxiety and/or fear was calculated for each individual and timepoint, and the means of the treatment groups were compared to each other. The owners also assessed the onset and end of any change or signs of effect in the cat’s behavior. Additionally, they assessed the usability of the product utilizing a scale of “very easy”, “easy”, “somewhat difficult” and “very difficult”.

### 2.6. External Expert Assessments

An external expert observer, blinded to the study treatment and owner assessment, evaluated the treatment effect during transportation based on video recordings using the same 5-point scale as the owner. Additionally, the external observer assessed the signs of stress, anxiety, and/or fear during transportation according to the frequency and/or duration, depending on the type of behavior (Table 5).

### 2.7. Safety Assessments 

As safety variables, the investigator assessed the alertness (physical and mental activity) and potential sedation of the cat at the beginning of clinical examination. Additionally, the owner scored at home the cat’s activity and ability to stand up and walk when opening the carrier, and 1 and 3 h after coming home (Table 6). Blood samples were collected at screening and treatment visits for hematological (e.g., blood cell counts, anaemia parameters, and white blood cell differential) and clinical chemistry (e.g., kidney and liver parameters, and electrolyties) analysis, and adverse events were recorded throughout the study.

### 2.8. Statistics 

Sample size was estimated based on a previously conducted pilot study [14]. The estimated sample size in each group was 81, with a 5% level Chi-square test having a 90% power to distinguish between an active treatment group and placebo. It was assumed that variation would be larger in this study than in the pilot study due to several centers, countries, and possible dropouts. Thus, at least 90 cats were to be recruited for both the pregabalin group and the placebo group by the end of the study.

Both primary variables were analyzed with a generalized linear mixed model appropriate for a multinomial response variable, with a cumulative logit link function. Treatment was modeled as a fixed effect, and center and center-by-treatment interaction as random effects. The baseline score was included as a covariate. As a supportive analysis, both primary variables were also dichotomized into success/failure variables following the predefined plan, where “success” was defined as “excellent” or “good” in the 5-point rating scale. All other scores (“fair”, “poor”, “very poor”) were regarded as “failure”. Dichotomized variables were analyzed with a logistic regression model. The inter-rater reliability between owners and external observer assessments of treatment effect during transportation was assessed with Cohen’s weighted kappa coefficient (*κ*).

The multinomial secondary variables were analyzed with a similar model as the primary variables. Change from baseline in owner’s assessment of sum of signs of stress, anxiety, and/or fear was analyzed with a linear mixed repeated measures analysis of covariance (RM-ANCOVA) model. Treatment, time and treatment-by-time interaction were fixed effects and subject, center and center-by-treatment interaction were random effects. Estimates for individual time points were done using contrasts. External observer assessment of signs of stress, anxiety, and/or fear were analysed descriptively.

All safety variables were reported descriptively by the treatment group. Differences were considered to be statistically significant with *p* < 0.05. All randomized cats (intention to treat [ITT] population) were included in the safety analysis. As the aim was to study the anxiolytic effect of pregabalin, a conservative approach was chosen where the cats were excluded from ITT population in the efficacy analyses if signs of sedation were seen. The predefined criteria in the study protocol stated that cats showing sedation at the clinic when evaluated at the beginning of clinical examination, or cats that were very calm/sleeping and showed moderate or severe incordination at two timepoints after coming home were excluded from the efficacy analysis. 

## 3. Results

### 3.1. Animals

A total of 214 client-owned cats entered the study. Five of them discontinued the study before treatment administration, thus 209 cats (one from Germany, 18 from Ireland, 23 from Finland, 57 from Hungary, and 110 from Portugal) were randomly allocated to receive either pregabalin 5 mg/kg (n = 108) or placebo (n = 101). The mean (SD) actual dose of pregabalin was 5.6 (5.3) mg/kg, dose volume was 0.5 (0.3) mL per cat, and the median (range) duration of car transportation was 22 (20–45) min and 22 (20–50) min for 5 mg/kg and placebo, respectively. The demographic and baseline characteristics were comparable in the treatment groups (Table 7).

### 3.2. Primary Efficacy Variables

**Figure 2:**
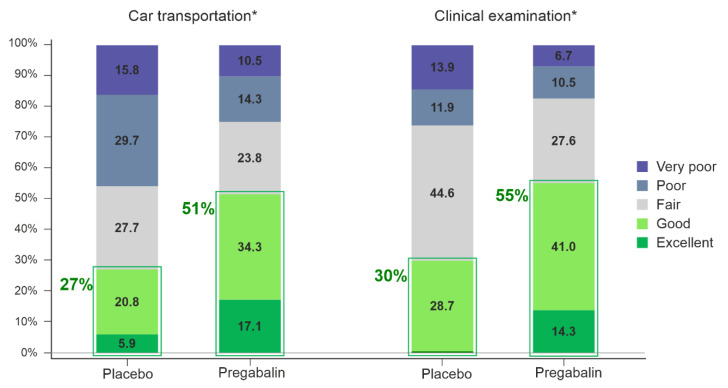
Distribution of responses (percentage of cats) for owner’s assessment of the treatment effect during transportation and investigator’s assessment of the treatment effect during clinical examination. * Indicates a statistically significant difference between pregabalin 5 mg/kg and placebo treatments.

A statistically significant difference favoring pregabalin 5 mg/kg over placebo was seen in both primary efficacy variables, the owner’s assessment of the treatment effect during transportation (OR 3.8 [95% CI 1.8–8.1], *p* < 0.01) and the investigator’s assessment of the treatment effect during clinical examination (OR 3.4 [95% CI 1.8–6.4], *p* < 0.01) (Figure 2). 

Cat owners assessed pregabalin to more often have ”excellent” or ”good” effect during the transportation in 51% (54/105) of cases compared to in 27% (27/101) of placebo cases (*p* < 0.01). The investigators’ assessment revealed more often ”excellent” or ”good” treatment effect during the clinical examination in cats treated with pregabalin, 55% (58/105), compared to cats receiving placebo, 30% (30/101), (*p* < 0.01).

### 3.3. Secondary Efficacy Variables

**Figure 3:**
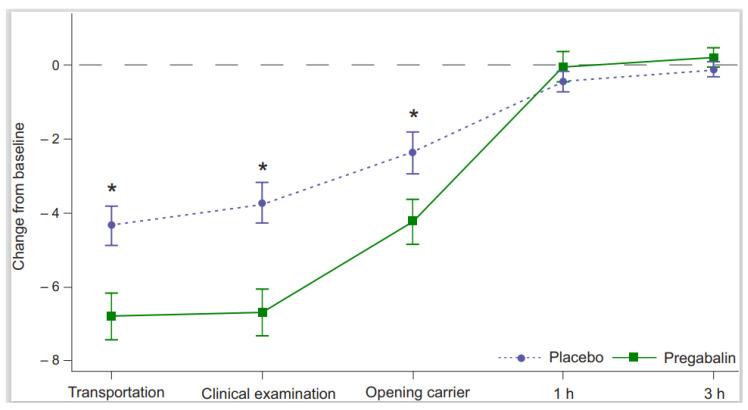
Mean (±SEM) change from baseline in the sum of signs of anxiety and/or fear during transportation, clinical examination, at home when opening the carrier, and 1 and 3 h after coming home. * Indicates a statistically significant difference between pregabalin 5 mg/kg and placebo treatments.

The treatment effect in the owners’ assessment of the ability to place the cat into the carrier was statistically significant favoring pregabalin over placebo (OR 6.0 [95% CI 2.0–17.9], *p* < 0.01). The mean sum of signs of anxiety and/or fear decreased from the screening visit with both treatments. The difference between pregabalin and placebo in the mean sum of signs at treatment visit was statistically significant during transportation (−2.9 [95% CI −4.3 to −1.5], *p* < 0.01), clinical examination (−2.8 [95% CI −4.2 to −1.5], *p* < 0.01) and when opening the carrier at home (−1.8 [95% CI −3.3 to −0.4], *p* = 0.02) favoring pregabalin (Figure 3). Based on the owners’ observations, vocalization, panting/intense breathing, resistance, and abnormal activity were the signs with the greatest numerical decrease with pregabalin treatment versus placebo. Owners were able to detect the onset and end of any change or signs of effect in 45% (49/108) of cats receiving pregabalin with the median duration of changes being 7 h (range of 1.3–28.5). The reported range is wide, mostly because some owners with the clinic visits taking place during evening hours evaluated the end of possible changes the next morning. 

The treatment compliance was good as 95% (103/108) of the pregabalin administrations were successful. Approximately 79% (81/103) of cat owners assessed that it was ”very easy” or ”easy” to administer the flavored pregabalin oral solution. 

### 3.4. External Expert Assessments

The external expert observer’s assessment of the treatment effect during transportation confirmed the owners’ assessment as pregabalin was statistically significantly better compared to placebo (OR 3.4 [95% CI 1.8–6.4], *p* < 0.01). The treatment effect was assessed ”excellent” or ”good” in 54% (54/101) of cats with pregabalin and in 43% (42/97) with placebo (*p* < 0.01). The owners’ and external observer’s agreement was moderate (*κ* = 0.47, *p* < 0.01). The external observer found the greatest numerical decrease in pregabalin treatment versus placebo in vocalization, dilation of pupils, flattening of ears, lip licking, and swallowing. In a total of eight cats, the lack of evaluable video material caused the absence of external observer assessment of these cats. Challenges with the video material in general included too dark of an environment during car rides taking place in the evening and technical problems with the video camera (e.g., running out of battery, missing audio, and incorrect focusing of the camera in the carrier). To mitigate these challenges observed early in the study, the owners were advised to use light colored bedding in the carrier, schedule the clinic visit to occur at daytime, if possible, and to increase attention on the function and the focusing of the video camera to improve video quality.

### 3.5. Safety

The investigators considered the majority of cats in both groups to have normal alertness. Only one cat in the pregabalin group was considered to show signs of mild sedation by the veterinarian and was thus excluded from the efficacy analysis. Based on the owners’ assessment, two additional cats were excluded from the efficacy analysis, according to the predefined criteria in the study protocol, as they scored to be both very calm or sleeping and having moderate incoordination at least in two time points after coming home. In the sensitivity analysis, when the three cats with mild signs of sedation were included, the efficacy results were similar to the ones in the main analysis.

The owners assessed a few more cats being very calm or sleeping in the pregabalin group (3%, 3/101) compared to placebo (1%, 1/87) after coming home from the veterinary clinic at the treatment visit. The ability to stand up and walk was assessed normal in 56% (57/101), 58% (59/101), and 73% (74/101) of cats in pregabalin group and 90% (78/87), 91% (79/87), and 95% (83/87) of cats in placebo group at home when opening the carrier and 1 and 3 h after coming home on treatment visit, respectively. 

There were few adverse events reported in the study, the most common being mild transient incoordination (five events in four cats) and tiredness (three events in three cats). These adverse events had resolved by the next day.

Seven cats in the pregabalin group and 14 cats in placebo group required sedation at the treatment visit approximately 2 h after administration of the study treatment to complete the standardized clinical examination including blood sampling. The sedatives used (e.g., alpha-2 agonists medetomidine, dexmedetomidine or xylazine, and opioids butorphanol or methadone), and their dosages were similar to those used at the screening visit for the concerned cats, and no safety concerns were reported. There were no notable changes in the laboratory values between the screening and treatment visits in either the pregabalin or placebo groups with the exception of one cat in each group. The clinically relevant findings, leucopenia in one cat in the pregabalin group and thrombocytopenia in one cat in the placebo group, were reported as adverse events. 

## 4. Discussion

The study results confirm that the novel pregabalin oral solution given at 5 mg/kg is effective in alleviating acute anxiety and fear associated with transportation and veterinary visits in cats, as both primary efficacy endpoints, the owner-assessed treatment effect during transportation and the investigator-assessed treatment effect during clinical examination, were met. Anxious cats were 3.8 times more likely (*p* < 0.01) to remain calm and quiet during transportation after treating with pregabalin approximately 1.5 h before the start of the car ride compared to the placebo. The veterinarians were 3.4 times more likely (*p* < 0.01) to easily perform the clinical examination after pre-visit medication with pregabalin compared to the placebo.

According to the baseline data, most cats entering the study had been highly distressed during transportation and clinical examination, as more than 67% of cats showed at least three severe signs of anxiety (i.e., present at least half of the time) at those timepoints at screening. A clear and clinically relevant treatment effect was confirmed by decreased signs of anxiety and fear during transportation and veterinary visit after pregabalin treatment based on the owners’ and external observer’s assessments. The signs with a numerically greatest change after pregabalin treatment, vocalization, panting, resistance, abnormal activity, dilation of pupils, flattening of ears, lip licking, and swallowing, are described in the literature as typical signs of stress and anxiety in cats [3,4,21,22,23].

The decrease in the sum of signs of stress, anxiety, and/or fear compared to the baseline was noted, especially during transportation and clinical examination and to a lesser extent also when opening the carrier after coming home. Based on earlier studies, it is known that these timepoints are very stressful for cats [1,2,3,4,5]. The signs of stress, anxiety, and/or fear were low in both frequency and extent at 1 h and 3 h timepoints after coming home from screening and treatment visits, most likely because the cats felt safe at home and were able to settle down after a stressful experience when in a comfortable and familiar place. This is contrary to the findings of an earlier study [5]. No statistically significant difference in the change from baseline of the mean sum of signs was seen between treatment and screening visits at 1 h and 3 h timepoints. A placebo effect was seen in the mean sum of signs during transportation, clinical examination, and when opening the carrier after coming home, and similarly also in the primary efficacy variables at the same timepoints. The placebo effect is a common phenomenon observed in double blinded placebo controlled studies in dogs and cats [19,24,25,26].

The owners and external expert used the same 5-point scale for assessment of the treatment effect during transportation. The calculated agreement between their assessments was highly significant. Owners generally may not recognise all the signs of stress in their cats to the same extent as a trained behavioural expert [4]. In addition, owners and experts observed the cat from different viewpoints as the owners know their own pet and might look at the cat subjectively while the external observer evaluates the cats more objectively, purely rating the cats behaviour. 

Pregabalin was well tolerated in cats, with mild and transient incoordination and tiredness as the most frequently reported adverse events. The owner assessments of the cats’ activity and ability to stand up and walk after coming home are generally in line with the safety findings. Allthough the owners noted some cats in the pregabalin group were very calm or sleeping after coming home, a similar trend could also be seen in cats treated with the placebo. This may be related to the finding that tiredness is a normal reaction in cats after stressful events and disruption of their normal daily routines [27].

Sedatives were used at the clinic, a decision made by the cat’s veterinarian to complete the annual exam, with informed consent and without safety concerns in seven cats after receiving pregabalin before the visit. This outcome suggests that healthy cats given a pre-appointment dose of pregabalin may be sedated during the following veterinary visit with commonly used sedatives, even though the number of cats sedated after a single dose of pregabalin is small. In this study, the doses of sedatives used at the treatment visit were similar to the doses used at the screening visit. However, as any central nervous system depressants may potentiate the effects of pregabalin, an appropriate dose adjustment should always be considered based on the clinical assessment. In humans, anxiolytic medicines are used as premedication in day surgery [28], and pregabalin was used safely to control both preoperative and intraoperative anxiety in patients undergoing anesthesia and surgery [29]. In cats, reduction of distress by applying a low-stress protocol during transportation to the veterinary clinic was shown to decrease the time to reach sedation and to reduce the required dose of an induction agent [30]. 

Pregabalin is currently approved in the European Union for use in humans for treatment of generalized anxiety, neuropathic pain, and epilepsy [31]. Recently pregabalin has been approved also in cats for alleviation of acute anxiety and fear related to transportation and veterinary visits [32] based on the results of this and other studies [14,16]. Currently, no other anxiolytic medicines are registered for travel- and veterinary-visit-related anxiety in cats. Gabapentin was studied and used in clinical practice to some extent [18,33,34,35] and there is one report of clinical use of trazodone [36]. Compared to gabapentin, pregabalin is a more potent molecule enabling similar efficacy with a much smaller dose. Pregabalin was reported to have more favorable pharmacokinetic properties with faster absorption and linear kinetic profile in humans [37]. Similar findings have also been reported in cats [15,16,38,39]. As the mode of action of both gabapentinoids is alike, their clinical efficacy and safety profile seem to be close to each other. However, both efficacy and safety depend on the used dose, which in cats is accurately studied and selected for pregabalin [40] but not so closely explored for gabapentin. 

Limitations of the present study include the lack of data on pregabalin use in cats with moderate or severe systemic diseases due to the inclusion criteria. This deficit leads to scarce information related to interactions with other medicines. Additionally, there was an absence of invaluable video material in eight cats due to practical challenges related to video recording during transportation, which may have complicated the external expert’s evaluations. Further limitations are that the study does not provide robust data regarding the duration of effect of pregabalin in anxious cats nor the pharmacokinetic data in pet cats for evaluation of anxiolytic plasma concentrations. In this study, owners were asked to record the onset and end of any change or sign of effect, which does not give reliable information on the duration of the actual anxiolytic effect. In general, owners were able to detect the onset and end of changes only for less than half of cats receiving pregabalin. Based on the limited data from the present study, it seems that the duration of effect of the novel cat-specific pregabalin formulation could be approximately 7 h. This estimate is supported by the pharmacokinetic parameters of pregabalin reported in cats [15,41], suggesting that cats have a higher degree of absorption and slower elimination compared to humans [42] and dogs [43]. More detailed studies are required to verify this finding.

## 5. Conclusions

The anxiolytic properties of the novel pregabalin oral solution with a dose of 5 mg/kg were measurable, statistically significant, and clinically relevant in cats with acute anxiety and fear associated with transportation and veterinary visits. The dose used was safe without a significant sedative effect. The owners found the cat-specific formulation with a small dosing volume easy to administer. The addition of this new product, with proven safety and efficacy in cats, provides practical aid for both owners and veterinarians for fear-free handling, and thus it improves the welfare of cats. 

## Figures and Tables

**Table 1:** Numerical rating scale for the owner’s assessment of the treatment effect based on the cat’s stress, anxiety, and/or fear during the transportation in a car. Modified from Lamminen et al. (2021) [14].

Score	Description
1	Excellent: Cat was calm and quiet during the whole transportation time, did not express signs of stress, anxiety, and/or fear.
2	Good: Cat was calm and quiet during most of the transportation time. Transient mild signs of stress, anxiety, and/or fear (e.g., occasional vocalization, salivation, or locomotion) up to 25% of the transportation time.
3	Fair: Cat showed moderate signs of stress, anxiety, and/or fear (e.g., vocalization, salivation, locomotion, or other activity in bouts) up to 50% of the transportation time.
4	Poor: Cat showed strong signs of stress, anxiety, and/or fear (e.g., vocalization, salivation, locomotion, or other activity almost without interruption or in longer, more forceful bouts) up to 75% of the transportation time.
5	Very poor: Cat showed extreme signs of stress, anxiety, and/or fear (e.g., vocalization, salivation, locomotion, or other activity forcefully and without interruption) for 75–100% of the transportation time.

**Table 2:** Numerical rating scale for the investigator’s assessment of the treatment effect based on the cat’s stress, anxiety, and/or fear during the clinical examination at the clinic. Modified from Mills et al. (2006) [17] and van Haaften et al. (2017) [18].

Score	Description
1	Excellent: Clinical examination could be easily performed without resistance or with insignificant resistance (no restraint needed). Cat was compliant and not frozen and did not express signs of stress, anxiety, and/or fear.
2	Good: Minor resistance; clinical examination could be performed with the technician minimally restraining the cat by placing a hand on the head or back. Cat was compliant and not frozen and expressed mild signs of stress, anxiety, and/or fear.
3	Fair: Moderate resistance or freezing. Cat expressed moderate signs of stress, anxiety, and/or fear. Clinical examination could be performed with the veterinary technician using physical restraint involving stabilizing the cat and holding in place. Freezing is defined as a moderately tense body.
4	Poor: Strong resistance or freezing. Cat expressed strong signs of stress, anxiety, and/or fear. Clinical examination could be performed without sedation with the veterinary technician more tightly restraining the cat (physically wrapping or scruffing cat). Freezing is defined as a very tense body, e.g., absence of movement except respiration.
5	Very poor: Extremely strong resistance. Cat expressed extreme signs of stress, anxiety, and/or fear and responded to the clinical examination with avoidant and/or defensive behaviour to an extent that completing the examination required sedation.

**Table 3:** Standardized clinical examination performed by the investigator at the veterinary clinic with specified order of performance in increasing invasiveness.

Order	Procedure
1	Cat carrier placed on examination table.
2	Carrier opened, and cat allowed to exit the carrier within approximately 1 min.
3	Cat removed from carrier, if it did not come out on its own, and placed on mat on the table. Assessment of alertness was done at the treatment visit.
4	Cat stroked dorsally on the table.
5	Heart and lungs auscultated. Heart rate and respiratory rate recorded.
6	Head held, eyes checked visually (primarily without any device).
7	Head held, ears checked visually (primarily without any device).
8	Head held, external neck and salivary glands and lymph nodes examined.
9	Head held, lip lifted – gums checked – capillary refill time (CRT).
10	Head held, mouth opened and checked.
11	Cat’s neck held – prescapular lymph nodes checked, hand ran down cat to check skin, popliteal lymph nodes checked.
12	While cat is standing belly palpated.
13	Proprioception checked for front and hind limbs.
14	Tail lifted, anus visualised.
15	Rectal temperature measured.
16	Blood sampling.

**Table 4:** Numerical rating scale for the owner’s assessment of the extent of signs of distress, anxiety, and/or fear. Modified from Lamminen et al. (2021) [14].

Score	Description
0	None
1	Only a few times
2	Half of the time
3	Most of the time
4	Continuously

**Table 5:** The signs assessed by the external observer in video assessment of cats. Modified from Lamminen et al. (2021) [14].

Signs Assessed by Frequency	Signs Assessed by Duration
Elimination	Crouched position
Eyes closed	Ears flattened
Lip licking	Exploration
Purring events	Grooming
Shake off	Hiding
Swallowing	Locomotion
Vomiting	Pupils dilated
Yawning	Purring
	Panting
	Scratching
	Salivating
	Sleeping
	Tail close to body
	Vocalization
	Withdraw
	Other

**Table 6:** Numerical rating scale for the owner’s assessment of the cat’s activity, and ability to stand up and walk. Modified from Korpivaara et al. (2017) [19] and Korpivaara et al. (2022) [20].

Score	Description of the Cat’s Ability to Stand up and Walk	Description of the Cat’s Activity
1	Normal: Cat is able to stand up and walk normally.	Active: e.g., mobile, pacing, trying to evade or hide when approached, hissing, crying, growling.
2	Calm: e.g., cat is able to stand up, can walk almost normally once it is moving although it may move more slowly. Walk may involve occasional mild staggering/incoordination.	Neutral: e.g., attentive, walking, standing, sitting normally, or normally lying down, behaves as usual.
3	Mild incoordination: e.g., cat can stand when encouraged or lifted up, mild staggering/incoordination when walking.	Calm: e.g., sitting or lying down, snoozing, may or may not look at observer, reacts to touch.
4	Moderate incoordination: e.g., cat can stand when encouraged or lifted up, hesitates to move, walking involves clear staggering/incoordination, may fall down when walking.	Very calm/sleeping: e.g., lying down or curled up, ignoring observer, eyes closed, does not react to touch or stimulation (e.g., when lifted up).
5	Severe incoordination: Cat is unable to stand up and walk.	

**Table 7:** Demographic and baseline characteristics.

Variable	Pregabalin(n = 108)	Placebo(n = 101)	Total(n = 209)
Sex, n (%)			
Male	51 (47)	36 (36)	87 (42)
Female	57 (53)	65 (64)	122 (58)
Age (years)			
Mean (sd)	5.3 (3.8)	5.7 (3.4)	5.5 (3.6)
Median (range)	4.7 (0.4–14.9)	5.5 (0.6–15.6)	4.9 (0.4–15.6)
Weight (kg)			
Mean (sd)	4.1 (1.2)	4.3 (1.2)	4.4 (1.2)
Median (range)	4.1 (2.3–7.6)	4.3 (2.1–10.3)	4.2 (2.1–10.3)
Neutered, n (%)			
Yes	87 (81)	87 (86)	174 (83)
No	21 (19)	14 (14)	35 (17)
Signs of severe anxiety at baseline (at least 3 signs at least half of the time), n (%)			
During transportation	76 (72)	74 (73)	150 (73)
At the veterinary clinic	76 (72)	68 (67)	144 (70)

## Data Availability

The data presented in this study are available on request from the corresponding author.
